# HA-coated collagen nanofibers for urethral regeneration via in situ polarization of M2 macrophages

**DOI:** 10.1186/s12951-021-01000-5

**Published:** 2021-09-22

**Authors:** Yuqing Niu, Florian J. Stadler, Xu Yang, Fuming Deng, Guochang Liu, Huimin Xia

**Affiliations:** 1 Department of Pediatric Surgery, Guangdong Provincial Key Laboratory of Research in Structural Birth Defect Disease, Guangzhou Women and Children’s Medical Center, Guangzhou Medical University, Guangzhou, 510623 Guangdong China; 2 Nanshan District Key Lab for Biopolymers and Safety Evaluation, Shenzhen Key Laboratory of Polymer Science and Technology, Guangdong Research Center for Interfacial Engineering of Functional Materials, College of Materials Science and Engineering, Shenzhen University, Shenzhen, 518055 Guangdong China

**Keywords:** Hyaluronic acid, Collagen, Nanofiber, M2 macrophage, Hollow organs regeneration

## Abstract

In situ tissue engineering utilizes the regenerative potential of the human body to control cell function for tissue regeneration and has shown considerable prospect in urology. However, many problems are still to be understood, especially the interactions between scaffolds and host macrophages at the wound site and how these interactions direct tissue integration and regeneration. This study was designed to evaluate the efficacy of hyaluronic acid (HA) functionalized collagen nanofibers in modulating the pro-healing phenotype expression of macrophages for urethral regeneration. Tubular HA-collagen nanofibers with HA-coating were prepared by coaxial electrospinning. The formation of a thin HA-coating atop each collagen nanofiber endowed its nanofibrous mats with higher anisotropic wettability and mechanical softness. The macrophages growing on the surface of HA-collagen nanofibers showed an elongated shape, while collagen nanofibers’ surface exhibited a pancake shape. Immunofluorescence and ELISA analysis showed that elongation could promote the expression of M2 phenotype marker and reduce the secretion of inflammatory cytokines. In vivo experiments showed that tubular HA-collagen nanofibers significantly facilitate male puppy urethral regeneration after injury. In the regenerated urethra bridged by tubular HA-collagen nanofibers, anti-inflammatory M2 macrophages are recruited to the surface of the scaffold, which can promote angiogenesis and endogenous urothelial progenitor cell proliferation.

**Supplementary Information:**

The online version contains supplementary material available at 10.1186/s12951-021-01000-5.

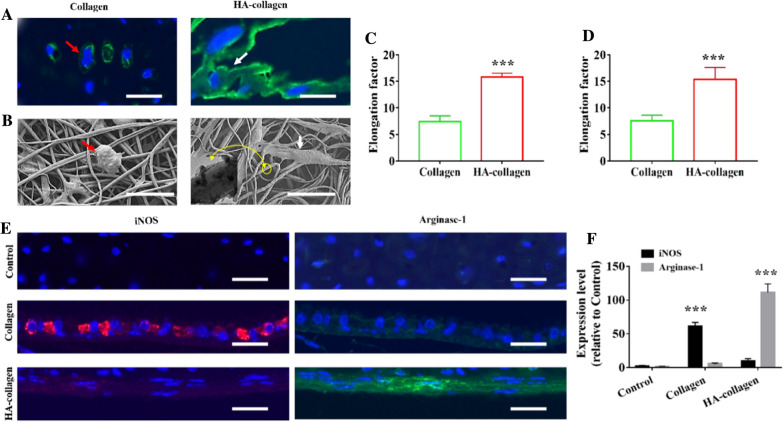

## Introduction

Regenerative medicine (RM) of tubular or hollow organs is a developing field of multi-disciplinary including materials science, engineering, biomedical and clinical research [1]. The majority of the hollow RM methods use scaffolds as a temporary extracellular matrix (ECM) function to guide tissue regeneration and remodeling, including the gastrointestinal tract [2], blood vessels [3, 4], respiratory system [5], and urogenital system [6, 7]. Collagen is the main structural element of natural ECM. Collagen is widely used as the scaffold matrix of tubular RM because of its good biocompatibility and easy processing [8–10]. The collagen fiber scaffolds produced by electrospinning technology have similar micro-architecture to ECM, and they have successfully reconstructed organs such as blood vessels and genitourinary tract in preclinical evaluation [2, 3, 6]. However, collagen nanofibers usually have limited ability to regulate capillary formation, epithelialization, and immune escape.

Similarly, as an ECM component, Hyaluronic acid (HA) plays a multi-faceted role in cell migration, proliferation, and differentiation at the micro-level and system-level events such as tissue water homeostasis [11, 12]. Recent studies have shown that one of the HA’s biological functions is to interact with cytokines and contribute to their retention in the ECM microenvironment [13, 14]. In addition, a specific reason for the increased use of HA-based scaffold structures is their immune regulation and regeneration induction abilities [15, 16]. Therefore, it is feasible to immobilize HA on the surface of collagen nanofibers to modulate cell reactions to promote the histocompatibility and epithelization of hollow tissue-engineered scaffolds.

Although progress has been made in tissue engineering and regenerative medicine, and it has been observed that HA and collagen-based composite nanofibers show the greatest vascularization and tissue regeneration in vivo, the regenerative biochemical cascade behind them has not been further explored [17–19]. Controlling the interaction between macrophages and biomaterials is very important to promote tissue regeneration in regenerative medicine. Due to their plasticity, macrophages are susceptible to environmental stimuli and play a double-edged sword role in the process of tissue regeneration in vivo [20]. Usually, macrophages can polarize into pro-inflammatory (M1) and pre-healing (M2) phenotypes. Some studies have shown that the polarization state of macrophages is related to the change of cell shape [21, 22]. Compared with the pancake-like M1 cells, cell elongation induced macrophage polarization to the M2-phenotype. In addition, elongation enhanced the effect of M2-induced cytokines and inhibited M1-induced cytokines, indicating that cell shape plays an essential role in regulating macrophage phenotype polarization [23]. Therefore, the development of RM scaffolds regulating macrophage elongation may promote tissue regeneration and regulate hollow organs’ structural remodeling.

Here, we demonstrate the epithelization efficiency of tissue-engineered HA-collagen fibers in male beagle puppies’ urethral wound site while shifting macrophage polarization. Compared with pure collagen nanofibers, HA-collagen nanofibers can induce macrophage elongation in vitro and in vivo. Elongation can enhance the expression of M2 phenotype marker arginase-1 and reduce the secretion of M1 phenotype marker. We also carried out detailed in vivo functional, retrograde cystourethrography and histological experiments to study the tissue-engineered tubular scaffold’s effect on the urethral structural remodeling at the wound site of the host urethra.

## Experimental section

### Materials

Hyaluronic acid (M_w_ ≈ 80 kDa, MB3113) and Collagen (MB5213) were obtained from Meilunbio®. Dulbecco’s modified Eagle’s medium (DMEM)/F-12 (10,565,018), DMEM (10,567,022), fetal bovine serum (FBS, 12,483,020), penicillin/streptomycin (P/S, 10,378,016), trypsin (0.25 %, 15,050,057), phosphate-buffered saline (PBS, pH 7.4, 10,010,023), Live/dead assay kit (L3224), diamidino-2-phenylindole (DAPI, 62,248), Alexa Flour™ 488 Phalloidin (A12379), primary antibodies to arginase-1 (PA5-85267), nitric oxide synthase (iNOS, PA3-030 A), keratin5 (K5, MA5-14473), CD34 (MA5-18091) were obtained from Gibco. Collagenase IV(C5138), bovine serum albumin (BSA, B2064), paraffin (1,496,904), and dehydrated alcohol (PHR1070) were obtained from Sigma, 100 μm cell strainer (352,350, BD Falcon), 4 % paraformaldehyde (C104190, Aladdin®), Triton X-100 (WGT8200), hematoxylin (HE, G1004), eosin (G1002), Masson’s trichrome stain kit (C1006), Verhoeff van Gieson stain kit (VVG, GP1054), and donkey anti-rabbit immunoglobulin-G (IgG) cy3- (GB21403) were obtained from Servicebio Science & Technology Co., Ltd. Primary antibodies to α-smooth muscle actin (SMC) (ab5694), CD31 (ab24590), and Ki67 (ab15580), and goat anti-rabbit IgG Alexa Fluor® 488-conjugated (ab150077) for fluorescence staining were purchased from Abcam. Hexafluoroisopropanol (HFIP, 920-66-1) and trifluoroethanol (TFE, 132248-58-9) were purchased from Aladdin. Mouse monocyte macrophages of cell line Raw 264.7 were obtained from Procell Life Science & Technology Co., Ltd.

### Preparation of HA-collagen nanofibers

Collagen (9 w/v%) in HFIP and HA (2.5 w/v%) in TFE were separately prepared as the core and the shell solutions, respectively. Then two syringes were used to fix the core and shell solutions on the precise high-pressure injection pumps (KDS100, KD Scientific). The coaxial electrospinning was carried out with concentric spinnerets. The inner diameter of the outer needle was 1.2 mm, and that of the inner needle was 0.3 mm. The injection speed of the shell solution injection pump is 2.5 mL/h, and that of the core solution injection pump is 0.11 mL/h.

The positive voltage is set at 12.1 kV, and the collector is a stainless steel needle with an outer diameter of about 2.6 mm and 200 mm length (for collecting tubular supports) or a 150 mm stainless steel rod (for collecting nanofiber films). The distance between the tip of the spinneret and the collector was set to be 150 mm. The speed of the collector is set to 1000 rpm. The environmental conditions are 18–25 °C and 45 % (relative humidity). The spinning process of collagen nanofibers is as described previously, the as-prepared 9 w/v% collagen solution is fixed on the high-pressure precision injection pump, and the electrospinning is carried out with a 22 G single needle spinneret. The injection speed of the solution injection pump is 0.27 mL/h, and the positive voltage is set at 12.1 kV. The collector is the same as that of HA-collagen nanofibers.

The obtained collagen and HA-collagen nanofiber films and catheters were crosslinked by glutaraldehyde (0.1 mol/L) and hydrochloric acid (0.01 mol/L) in acetone (80 %) water mixture at 25 °C for 24 h. After crosslinking, they were dried in a vacuum drying oven, packed into independent packaging bags, and then sterilized by 12 kGy Co^60^ irradiation for 30 min.

### Characterization of HA-collagen nanofibers

The morphology of electrospun nanofibers was investigated using a field emission scanning electron microscopy (SEM, SU8010, Hitachi) at an accelerating voltage of 3–5 kV. The average diameter and pore size of the electrospun fibers were measured from the SEM images by the ImageJ software package (https://imagej.net/citing). For each sample, an average number of 70 nanofibers was randomly counted using the ImageJ program [24]. The detailed comparative surface morphology of collagen and HA-collagen nanofibrous mats were observed using atomic force microscopy (AFM, MultiMode8, Bruker, Germany).

The HA-collagen nanofibers with partially washed HA-coating were transferred to copper mesh and dried *in vacuo* at room temperature (RT) for 24 h. The electrospun HA-collagen with HA-coating structure was studied by transmission electron microscopy (TEM, Tecnai G2 20, Hillsboro, US).

Fourier transform infrared spectroscopy (FTIR) with an attenuated total reflectance (ATR) head (ATR-FTIR) of the collagen and HA-collagen nanofibers were characterized by Nicolet IR 200 (Thermo Electron, USA) spectrophotometer.

Static water contact angles of the electrospun nanofibers were measured by the sessile method at room temperature under an air atmosphere using a contact angle analyzer (JC2000D1, POWEREACH, Inc.). Briefly, the de-ionized water (2 µL) was dropped onto a flat nanofiber film, and a static image was taken. Then the contact angle of water was measured in two directions parallel to the nanofiber axis (*n* = 5).

Electrospun nanofiber films were cut into 20$\times$6 mm^2^ rectangles with a thickness of 80–110 μm, which was used for further calculations while ignoring the porosity inherent to electrospun mats. The tensile strength measurements were then tested using an Instron mechanical testing machine (AG-IC50kN, Shimadzu, Inc.), equipped with a 100 N load cell and pre-set at a clamp distance of 1.4 cm. Tensile stress, fracture strain, and Young’s modulus were measured at a 1 mm/min constant crosshead speed until failure (*n* = 5). Owing to the porous nature of the electrospun nanofiber films, however, stress and modulus are underestimated on a material level, while it is expected that the measured strain at break is higher than a bulk material of equal chemical structure.

### Cell experiments

#### Cell culture

Raw 264.7 macrophages were seeded at 1.0$\times$10^4^ cells per well in sterile collagen or HA-collagen nanofibrous thin film (φ = 13 mm, 20 μm thickness) coated 24-well plates and cultured for 48 h at 37 °C with an atmosphere of 5 % CO_2_ in maintenance medium: DMEM/F-12 with 10 % v/v FBS and 1 % v/v P/S. Cells on the nanofibers were fixed with 4 % paraformaldehyde for 15 min at RT. The 4 % paraformaldehyde was then removed carefully and washed twice with PBS. After the freeze-drying oven is dried overnight, images of cell morphologies on nanofibers were captured by SEM.

#### Cytoskeleton F-actin staining

To evaluate the morphological changes of macrophages, the cells were cultured on collagen or HA-collagen nanofibrous films and then stained with Alexa Flour™ 488 Phalloidin. In short, after fixation with 4 % paraformaldehyde for 10 min, washed thoroughly with PBS, and then cultured in Alexa Flour™ 488 Phalloidin (5 µg/mL) for 30 min. The nuclei were counterstained with DAPI at a concentration of 1.4 µg/mL for 10 min, and samples were examined by inverted fluorescence microscopy (DMi8, Leica) [25].

#### Cell morphology analysis

As described previously, 6 random SEM or fluorescence staining micrographs were selected by ImageJ software for quantitative analysis of cell elongation. The elongation index was calculated by the ratio of the long axis to the short axis, which was used to evaluate the morphological changes of macrophages [23].

#### Live/dead assay

Raw 264.7 macrophages at a density of 1$\times$10^4^ cells were seeded into the HA-collagen and collagen nanofibrous film (0.5 cm in length) coated dishes, respectively, for 24 h. They were transferred into new 96-well plates and cultured in DMEM supplemented with 10 % FBS at 37 °C with an atmosphere of 5 % CO_2_. On day 7, the abovementioned cell-seeded nanofibrous films were stained using a live/dead assay kit. Briefly, the cell-seeded nanofibrous films were washed with 1$\times$ PBS once and then incubated for 30 min in the staining solution that contained 0.4 µL of Calcein AM and 2 µL of ethidium-homodimer-1 in 1 mL of 1$\times$ PBS. The cells seeded nanofibrous films staining was observed using a confocal laser scanning microscope (CLSM, SP5, Leica, German), where the excitation/emission filters were set at 488/530 nm to observe living cells (stained green) and at 530/580 nm to detect dead cells (stained red). Raw 264.7 macrophages grown into the 96-well cell culture plate were set as a positive control. All the samples were tested in triplicate. Five random views from each nanofibrous film were photographed and analyzed.

### Immunofluorescence staining

To evaluate the phenotypic polarization of macrophages in different nanofibrous films, immunofluorescence staining of iNOS or arginase-1 was performed. After being cultured in different nanofibrous films for 24, 48, and 72 h, then fixed with 4 % paraformaldehyde for 10 min, the samples were incubated in 1 % BSA containing 0.1 % Triton X-100 for 30 min at RT. Cells were treated with a mouse monoclonal antibody to iNOS or arginase-1 at a 15 µg/mL concentration for 4 h at 4 °C. The cells were then treated with a donkey anti-rabbit IgG cy3- or goat anti-rabbit IgG Alexa Fluor® 488-conjugated secondary antibody at a concentration of 2 µg/mL for 45 min at RT. DAPI was used to stain the cell nuclei. Fluorescence micrographs were captured using a Leica TCS SP5 CLSM. Histomorphometric evaluations were performed on 6 independent microscopic fields using ImageJ software to quantify the iNOS or arginase-1 positive expression. For each type of sample, 6 random images were selected for quantification at 200× magnification.

### Western blotting

To analyze the macrophage phenotypic shape markers arginase-1 and iNOS, the cells cultured on each nanofiber membrane sample were separated by 0.25 % trypsin and transferred to the lysis buffer containing 1 % benzenesulfonyl fluoride; after 30 min of complete lysis at 4 °C, protein samples were collected by centrifugation at 25,000 rpm for 15 min, and the total protein concentration was measured by commercial kits. For Western blotting, the main antibodies used were anti-arginase-1 (1:1000, PA5-85267, Gibco), anti iNOS (1:1000, PA3-030a, Gibco), and anti-α-tubulin (1:1000, ab176560, Abcam), the subsequent procedures were performed as described previously [26–28].

### Cytokine secretion

To investigate the effect of different nanofibers on the polarization of macrophages, the secretion of cytokines was detected by ELISA. 264.7 macrophages (1.0$\times$10^4^ cells) were seeded in collagen and HA-collagen nanofibrous films or 24 well plates as the control group. The supernatant was collected, centrifuged, and used for ELISA after 24, 48, and 72 h. According to the manufacturer’s protocol, TNF-α and IL-10 were quantified by ELISA Kit (FEK0527, EK0417, Boster).

### Model of urethral defect

All in vivo experimental procedures involving animals in this study were conducted under Institutional Guidelines for Animal Care and approved by the Animal Ethics Committee of Guangzhou Medical University (Guangzhou, China). 12 male Beagle puppies at the age of 15 months at the start of the experiments were used. The animals were divided into 2 groups, each with 6 puppies. As described previously, [29] general anesthesia was induced and maintained with 45 mg/kg sodium pentobarbital (P0500000, Guangzhou pharmaceutical Medicine Co., Ltd.). Surgical disinfection was performed with Iodophor and 70 % ethanol. A sterile 6 F catheter was inserted into the bladder. A 3 cm skin incision was performed just proximal to the glans and the urethra within the corpus spongiosum was isolated. A 5.0 suture (VCP311H, Johnson Ames) was placed at the proximal and distal ends of the urethra. A 2.2 cm long urethral tissue was excised 0.5 cm from the bottom of the glans to form a urethral defect (Additional file 1: Fig. S1A). A 2.2 cm-long HA-collagen or collagen tubular graft was loaded on the catheter and double end-to-end anastomoses were performed to the native urethra with 6.0 Johnson Ames sutures (Additional file 1: Fig. S1B). After the defect tissue was washed with sterile normal saline for autograft implantation, the recovered urethral tissue was reversed and sutured. Finally, the skin was sutured with Johnson Ames 5.0 interrupted sutures (Additional file 1: Fig. S1C). Analgesia with Carprofen (Y0000846, Mreda Co., Ltd.) 4 mg/kg/day was continued for 5 days. After the operation, each animal was raised in a single cage.

### Retrograde cystourethrography test

As described previously, [29] the animals were subjected to general anesthesia for macroscopic evaluation and monthly urinary cystourethrography (meglumine diatrizoate, M861408, 200 mg/mL, Guangzhou pharmaceutical Medicine Co., Ltd.). All images were collected with a Luminos Digital Radiography system (Allura Xper FD20, Philips, Netherlands). The diameter of the urethra was measured utilizing a scale. Knowing that the graft is sutured at 0.5 cm from the base of the glans, and its length is 2.2 cm. The position of the graft can be determined on the X-radiograph. The condition of urethral regeneration in the graft area can then be estimated.

### Histological and immunofluorescence staining of urethral grafts

The samples were fixed in 4 % paraformaldehyde for 12 h, and then embedded in paraffin. Section (4 μm) were stained with HE, Masson’s trichrome and VVG, respectively. The samples were visualized by a Pannoramic desktop microscope (Pannoramic 250, 3D Histech), and the representative visual field was obtained by CaseViewer software (version 2.3). For each type of implant, 6 random images were selected for quantification at 400× magnification.

Urethral tissue cytokine measurement procedures were performed as described previously [29].

For immunofluorescence staining of paraffin-embedded tissue sections, the sections were first de-paraffinized in xylene and then rehydrated in graded alcohol series, subjected to heat-induced epitope retrieval in 10 mM citrate buffer (pH 6.0), followed by the immunostaining protocol according to the Cellular immunofluorescence procedure. The following antibodies: rabbit anti-Ki67 polyclonal antibody, rabbit anti-arginase-1 polyclonal antibody, mouse anti-K5 monoclonal antibody, or mouse anti-CD34 monoclonal antibody was used at a dilution of 1:100. The secondary antibody, Cy3-or AlexaFluor-488-conjugated anti-rabbit or mouse IgG was used at a dilution of 1:2000. Micrographs were obtained using the CLSM. Histomorphometric evaluations were performed on five independent microscopic fields using ImageJ software to quantify the Ki67 positive expression. For each type of implant, 5 random images were selected for quantification at corresponding magnification.

### Statistical analysis

All numerical data in this study are expressed as means ± standard deviation (SD). Statistical analyses were performed using GraphPad Prism 5 software. Statistical significance was determined using Student’s t-tests between two groups or one-way ANOVA executed by a post hoc Tukey comparison among groups; *p < 0.05 was considered significant.

## Results and discussion

### Electrospun HA-collagen nanofibers

**Fig. 1:**
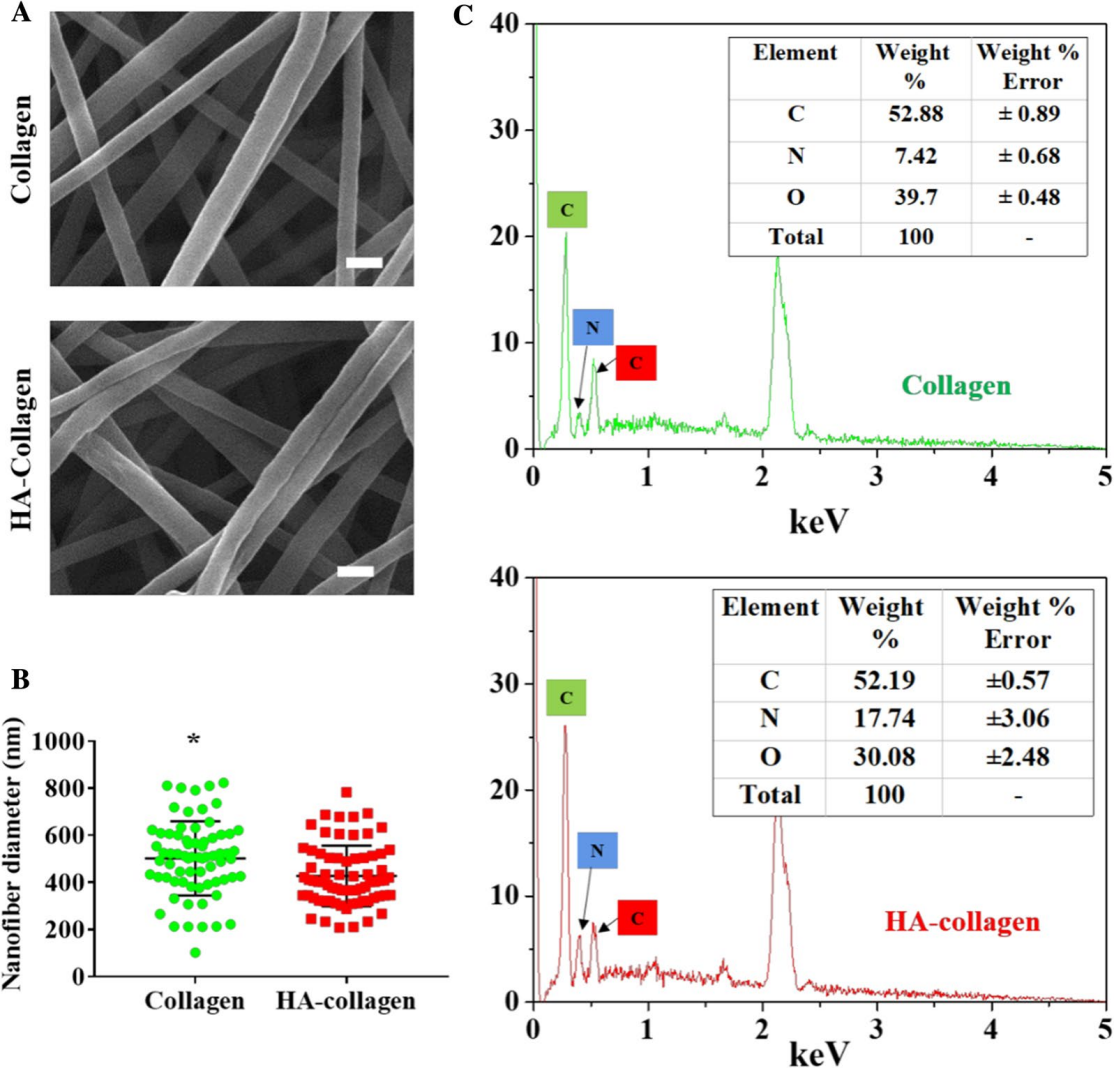
Surface nano-topography and chemistry distribution. **A** Representative SEM micrographs of collagen and HA-collagen nanofibrous films. Scale bars: 1 μm. **B** Statistical data of nanofiber mean diameter of each nanofiber sample. *n* = 70, *p < 0.05. (C) EDX spectra examination of the surface of collagen and HA-collagen nanofibers

SEM analysis showed that the surface morphology of HA-collagen nanofibers prepared by electrospinning was interconnected pore networks, similar to that of pure collagen nanofibers. The electrospun HA-collagen and collagen nanofibers are cylindrical nanofibers with good continuity (Fig. 1A). Quantitatively, the average diameter of HA-collagen nanofibers is (502$\pm$18) nm, slightly higher than that (427$\pm$15) nm of collagen nanofibers (Fig. 1B). The surface element distributions of Collagen and HA-collagen nanofibers were analyzed by energy-dispersive X-ray (EDX) (Fig. 1C). There is no significant difference in the distribution of elements on the surface of collagen and HA-collagen nanofibers. They were indicating that the HA-coating does not affect the surface chemical element distribution of collagen.

**Fig. 2:**
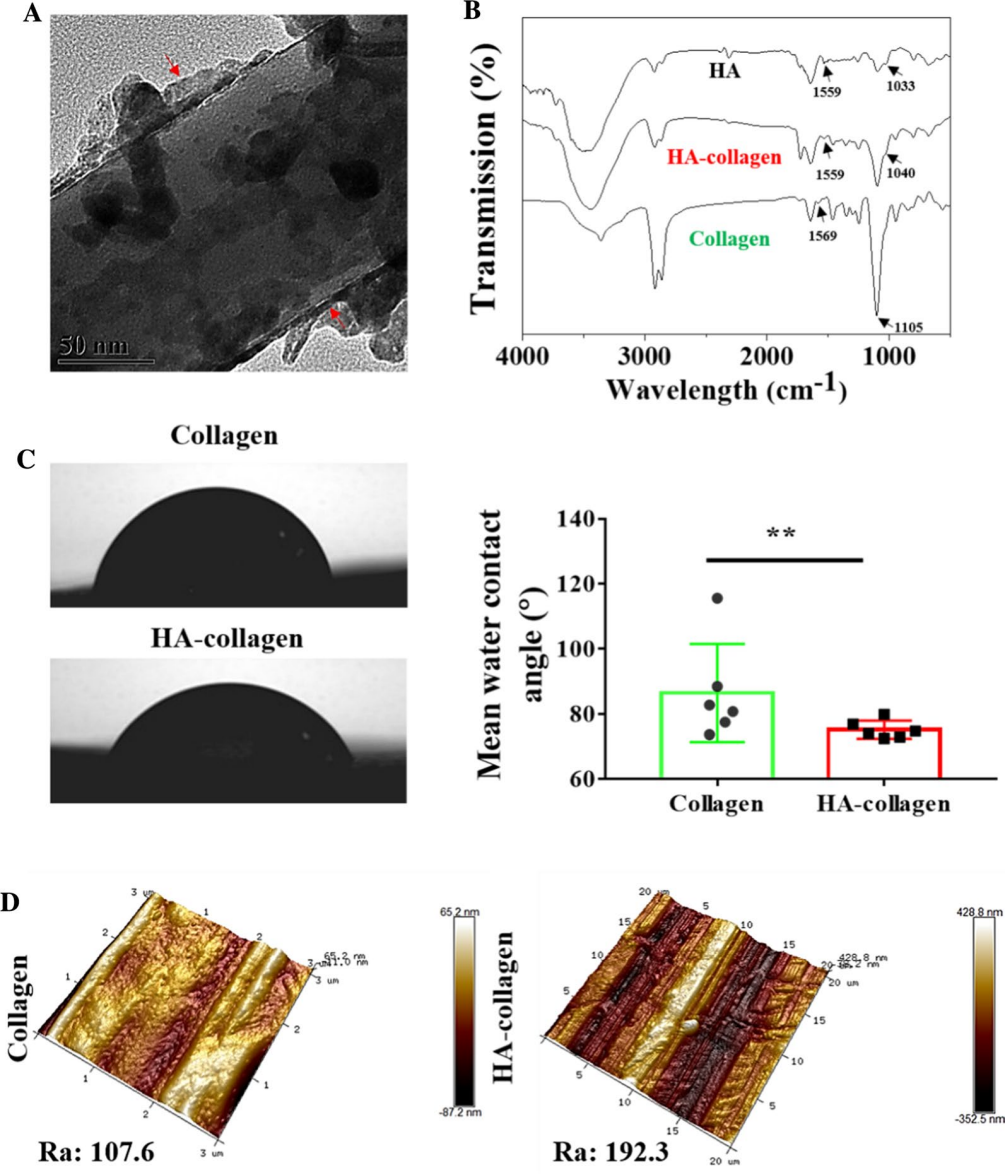
Surface biophysical characteristics. **A** Representative TEM micrographs of HA-collagen nanofiber. Red arrows indicate HA-coated collagen nanofiber. **B** ATR-FTIR spectra examination the surface of collagen, HA-collagen nanofibrous films and HA. **C** Histogram of the water contact angle data measured with the different nanofiber samples. Left panels are water contact angle photographs of the different nanofibrous films taken in the perpendicular and parallel directions to the fiber axis with a video microscope. *n* = 5, **p < 0.01. **D** High-resolution AFM surface morphology of collagen and HA-collagen nanofibrous films

To confirm that the hydrophilic polysaccharide polymer HA is encapsulated on the collagen core as a shell to form a HA-coating, the TEM, ATR-FTIR, and TGA analysis was employed. As shown in Fig. 2A, TEM micrograph clearly reveals the HA-coated collagen nanofiber. The coating thickness is estimated to be ~ 5 nm. Compared with collagen nanofibers, HA-collagen nanofiber has obvious absorption peaks at the wavelengths of 1559 and 1045 cm^− 1^, which may be due to the redshift caused by in-plane bending of amide N-H and Tensile vibration of C-N, indicating the existence of HA-coating (Fig. 2B). TGA curves of the collagen and HA-collagen nanofibers as well as HA are shown in Additional file 1: Fig. S2. Due to the decomposition of water and oxygen-containing groups adsorbed on collagen, the pure collagen nanofibers’ weight loss is 31.23 % at 500 °C, which is very similar to the HA-collagen nanofibers (31.5 %), which indicates that HA-coating was successfully bonded to the collagen nanofiber surface.

The static water contact angle analysis of electrospun nanofibers showed that the HA-collagen nanofibers had anisotropic wetting hydrophilicity, and their water contact angle was significantly lower than that of collagen nanofibers (Fig. 2C and Additional file 1: Table S1). This indicates that the improvement of HA-collagen nanofibers’ wettability is attributed to the hydrophilic effect of the HA coating. Figure 2D shows the high-resolution AFM images of the two kinds of nanofibers. Compared with pure collagen nanofibers, the average surface roughness (Ra) of HA-collagen nanofibers increases slightly. This may be due to the formation of a thin HA-coating layer atop each collagen nanofiber surface.

Both HA-collagen nanofibers and collagen nanofibers were tested in a uniaxial tensile mode to generate stress-strain curves and derived tensile properties (Additional file 1: Fig. S3). The ultimate tensile strength and Young’s modulus of collagen are (0.13$\pm$0.3) MPa and (0.35$\pm$0.3) MPa, respectively (Additional file 1: Table S1). Compared to collagen nanofibers, the ultimate tensile strength and Young’s modulus of HA-collagen nanofibers are close to Collagen nanofibers. However, the elongation at break is (101$\pm$28)%, which was slightly higher than that of collagen nanofibers (91$\pm$11)%, indicating that HA-collagen is a soft and tough substrate. Nevertheless, Young’s modulus of HA-collagen nanofibers is still higher than that of the native urethra (approximately 0.22 MPa) [10]. The above results showed that collagen nanofibers and HA-collagen nanofibers have similar viscoelasticity, stiffness, and surface chemical composition and roughness except for the significant difference in surface wetting.

### HA-collagen nanofiber pursued the polarization of macrophages to M2 phenotype in vitro

The surface properties of biomaterial scaffolds, such as hydrophilicity, softness, roughness, and chemical function, are critical to guide macrophage immune response [29–31]. Cell adhesion and diffusion is the first step in the interaction between cells and biomaterial scaffolds, which may regulate the behavior of immune cells, including polarization. Mouse monocyte macrophages of cell line Raw 264.7 were inoculated on the surface of collagen and HA-collagen nanofibrous thin films, respectively, for 12 h. Cell adhesion was evaluated by the CCK-8 method. The results showed that the surface properties did not affect the adhesion of macrophages (Additional file 1: Fig. S4).

**Fig. 3:**
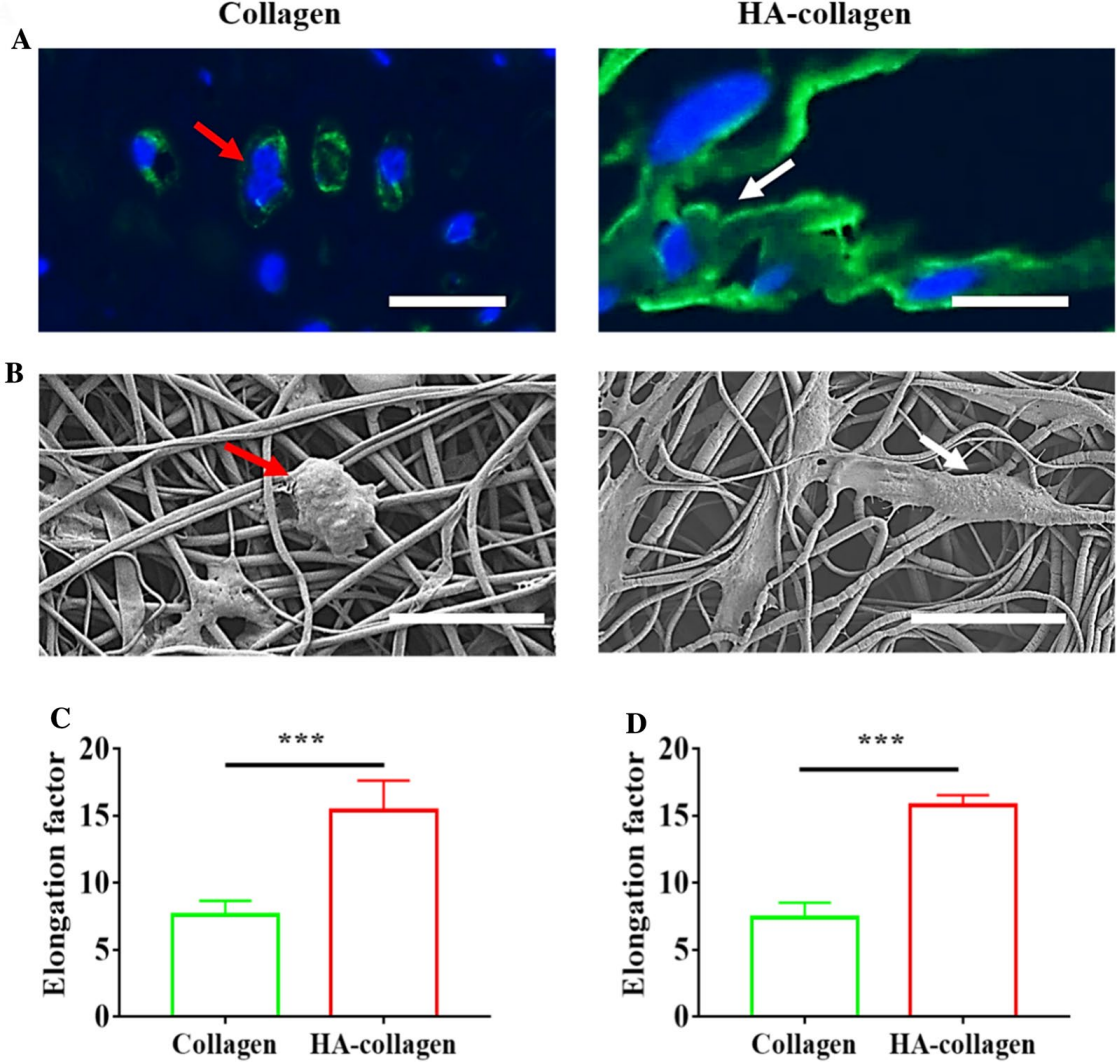
HA-collagen nanofibers promoted macrophage elongation in vitro. Representative macrophage shapes on collagen and HA-collagen nanofibrous films under Alexa Flour™ 488 phalloidin staining (**A**) and SEM (**B**). Red arrows indicate round, pancake-like shape macrophage, white arrows indicate elongated macrophage. Scale bars: (**A**) 20 μm; (**B**) 10 μm. Quantitative analysis of macrophage elongation based on images of Alexa Flour™ 488 phalloidin staining (**C**) and SEM (D). *n* = 3, ***p < 0.001

On the contrary, fluorescence microscopy analysis of cell spreading after 48 h of co-culture showed that the degree of elongation of macrophages on collagen nanofibers and HA-collagen nanofibers was very different: macrophages on collagen nanofibers were pancake-shaped, while cells on HA-collagen nanofibers were elongated (Fig. 3A). SEM images further confirm these phenomena (Fig. 3B). We also measured the elongation of macrophages growing on the surface of different nanofibrous films, and the elongation index was calculated as the length of the longest axis divided by the width across the nucleus [24]. The elongation index of macrophages growing on the surface of HA-collagen nanofibers was significantly higher than that of cells growing on the surface of collagen nanofibers (Fig. 3C, D).

**Fig. 4:**
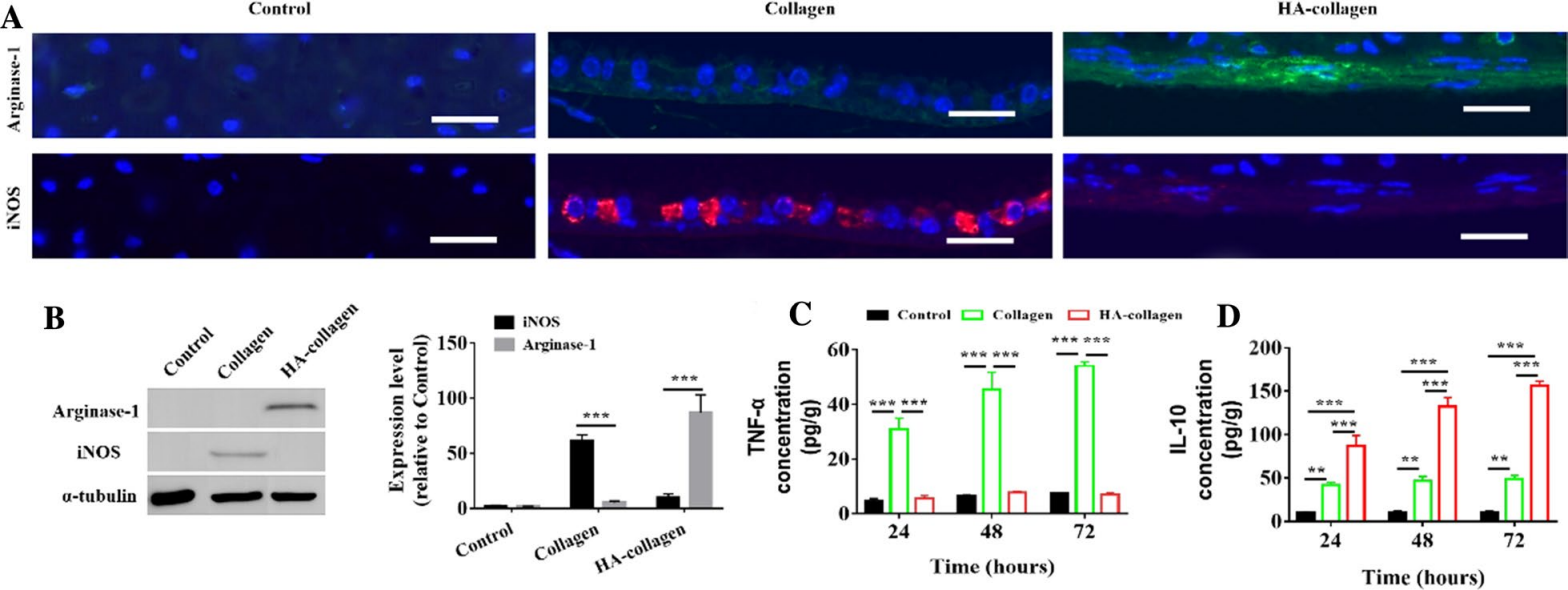
The polarization of macrophages to M2 phenotype is related to the elongated cell shape.** A** Fluorescence micrographs of Raw 264.7 macrophages immune-stained for arginase-1 (green), iNOS (red), and nuclear counterstain (blue) on cell plate (control), collagen and HA-collagen nanofibrous films. Scale bars: 15 μm. **B** Representative Western blot of arginase-1, iNOS, and tubulin of control, collagen and HA-collagen nanofibrous films and quantification of average across three separate experiments. Quantified TNF-α **C** and IL-10 **D** secretion from macrophages cultured on different nanofibrous scaffolds or culture plates using ELISA assay. *n* = 3, **p < 0.01, ***p < 0.001

To confirm the polarization status of macrophages, immunofluorescence staining and Western blotting were performed to evaluate the expression of iNOS and arginase-1, which are the specific markers of pro-inflammatory and pro-healing phenotypes, respectively [31]. We found that 264.7 macrophages grown on collagen nanofibers expressed high levels of iNOS, while 264.7 macrophages grown on HA-collagen nanofibers expressed higher levels of arginase-1 (Fig. 4A, B). Interestingly, we found that the pancake-like 264.7 macrophages expressed iNOS, while the elongated 264.7 macrophages expressed arginase-1. These results are consistent with the published reports that the polarization of macrophages to M2 phenotype is related to the elongated cell shape [20, 23, 32].

Studies have shown that the release of cytokines from M2 phenotype macrophages contributes to the recruitment of endogenous progenitor stem cells [30–32]. These endogenous cells synthesize and precipitate new proteins, which then penetrate the host for vascularization and functional recovery [33–35]. On the contrary, the release of cytokines by M1 phenotype macrophages impaired the recruitment of these endogenous cells [31]. Next, we studied whether the change of cell shape caused the change of cytokines released by macrophages. 264.7 macrophages were cultured on the surface of collagen nanofibers and HA-collagen nanofibers for 24, 48, and 72 h. IL-10 and TNF-α were detected by ELISA, which are mainly secreted by M2 and M1 macrophages, respectively. We found that compared with the cells cultured on collagen nanofibers, the cells cultured on HA-collagen nanofibers expressed significantly higher levels of IL-10, but lower levels of TNF-α (Fig. 4C, D). This is consistent with the established evidence that macrophage elongation stimulates M2 phenotype differentiation without affecting inflammatory activation [23, 30]. In addition, in vitro live/dead assay showed that collagen and HA-collagen nanofibers were biocompatible substrates and would not be toxic to macrophages growing on their surfaces (Additional file 1: Fig. S5). These results suggest that the HA-collagen nanofibers may be an ideal matrix to drive macrophages to polarize to the M2 phenotype.

### Tubular HA-collagen nanofibers recruit endogenous urothelial progenitor cells in wound site via in situ polarization of M2 macrophages

**Fig. 5:**
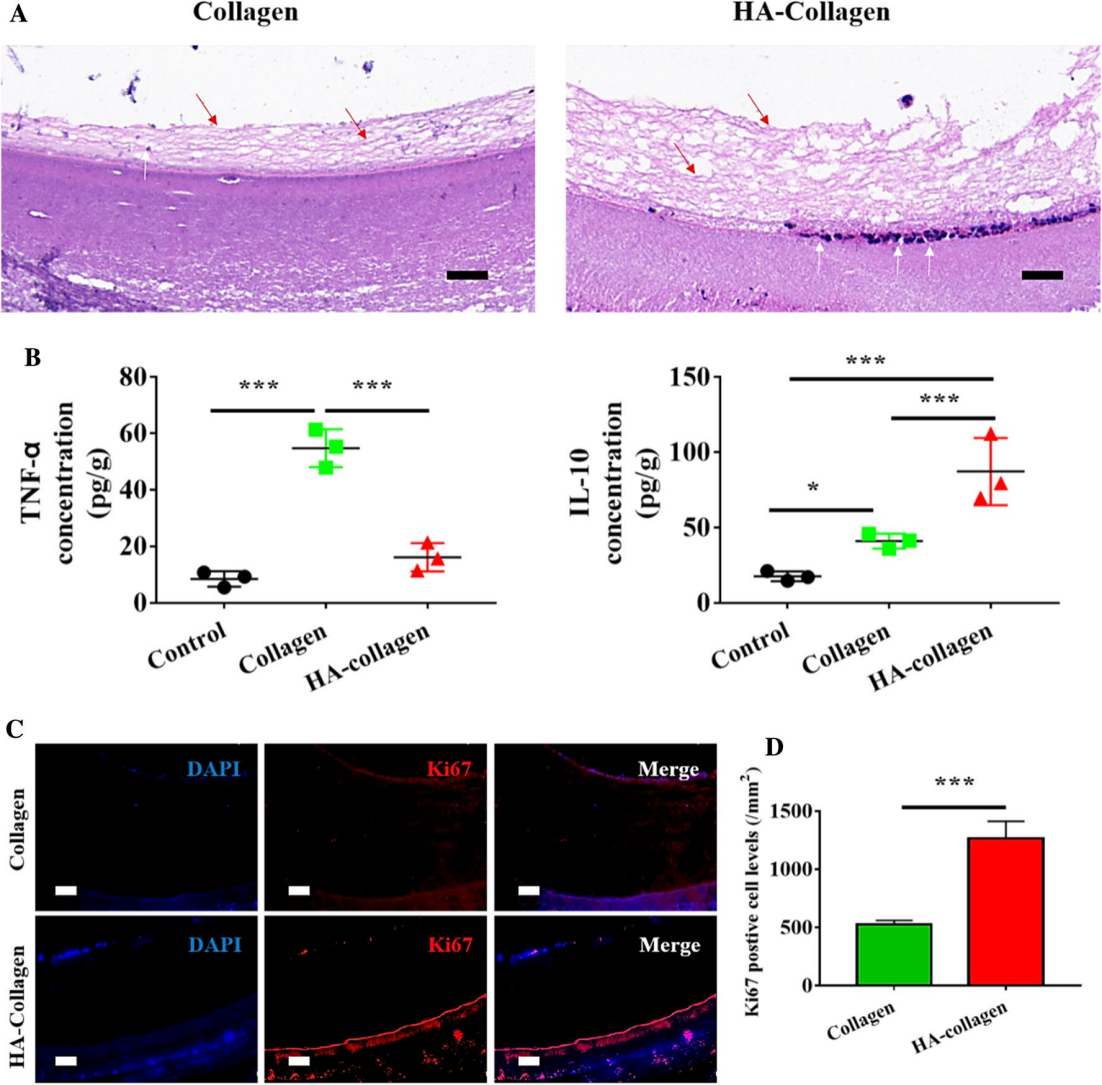
Tubular HA-collagen nanofibers enhanced endogenous cell recruitment and cell proliferation at wound site on week 2 after implantation. **A** HE staining the middle cross-section of regenerated urethra at 2 weeks after scaffold implantation. Scale bars: 20 μm. Red arrows indicate the ECM regenerated along the inner wall of the scaffold, and white arrows indicate the host macrophages collected along the inner wall of the scaffold. **B** The peri-implant concentrations of TNF-α and IL-10 when implanted at the wound site for 2 weeks. *n* = 3, *p < 0.05; ***p < 0.001. **C** Representative micrographs of Ki67 immuno-fluorescence staining of the two groups on week 2. Scale bars: 50 μm. **D** Statistical data of Ki67 positive cell levels of the two groups. *n* = 3, ***p < 0.001

Studies have shown that the phenotypic polarization of macrophages determines the immune response shortly after the implantation of biomaterial scaffolds. According to the surface biophysical and biochemical characteristics of biomaterial scaffolds, macrophages initiate pro-inflammatory or anti-inflammatory reactions [36–38]. The release of these molecules leads to the recruitment of endogenous progenitor cells and stem cells in scaffolds, which is the key step of in situ tissue regeneration [35, 39]. Due to the key role of M2 macrophages in situ tissue regeneration, we speculate that urethral grafts made of HA-collagen nanofibers may play a beneficial role in urethral tissue regeneration after injury. To test this hypothesis, we transected the urethra of male beagle puppies and bridged the resulting urethral gaps with tubular HA-collagen nanofibers. Tubular collagen nanofibers, a pervasive urethral graft, were used as the control group. Two weeks after the operation, the cross-sections of each graft were randomly taken for immunofluorescence of K5, a marker for urothelial progenitor cells [40], to observe the recruitment of endogenous cells. Compared with the collagen group, the lumen surface of HA-collagen bridged regenerated urethra was enriched with more elongated M2 macrophages (green), and were very close to one another, connected to the basement membrane by thin cytoplasmic extensions, which then led to more recruitment of urothelial progenitor cells (red) within the scaffold (Additional file 1: Fig. S6). The results showed more endogenous urothelial progenitor cells on the surface of the reconstructed urethral cavity bridged by tubular HA-collagen nanofibers. In addition, HE staining at low magnification showed that endogenous urothelial progenitor cells recruited on the inner surface of the two kinds of nanofibers synthesize and vertically deposit new extracellular matrix (loose network fibers on the inner wall of the scaffold). Compared with the collagen graft group, the HA-collagen graft group had higher ECM on the lumen of the regenerated urethra (Fig. 5A). It is consistent with the published report that the recruitment of endogenous progenitor stem cells determines the synthesis and deposition of new ECM on the surface of biomaterial scaffolds [20, 30].

Next, we investigated whether the recruitment of these endogenous cells along the surface of the graft lumen is related to M2 phenotype polarization. We detected the expression of IL-10 and TNF-α on the surface of the regenerated urethral lumen by ELISA. Similarly, 200 mg of tissue was removed from the urethra of beagle puppies who had never received a urethral graft as background cytokine control samples. The results showed that the expression of IL-10 in the grafted urethra bridged by tubular HA-collagen nanofibers was significantly higher than that in the tubular collagen nanofibers group (Fig. 5B), indicating that HA-collagen nanofibers can regulate macrophage polarization to M2 phenotype to guide the recruitment of endogenous urothelial progenitor cells. These results are consistent with previous reports that the accumulation of anti-inflammatory M2 macrophages along the surface of scaffolds is critical for the rapid recruitment, migration, and infiltration of endogenous progenitor stem cells at the wound site [41].

To study the effect of tubular HA-collagen nanofiber grafts on cell proliferation in vivo, the Ki67 immunofluorescence assay was carried out, which is a key marker of cell nucleus proliferation [42]. We found that a large number of Ki67 positive cells expressed red fluorescence in the tubular HA-collagen and collagen nanofiber grafts 2 weeks after transplantation (Fig. 5C). In the urethral graft fabricated with collagen and HA-collagen nanofibers, cells were located on both sides of the cross-section lumen of the scaffolds and covered more than 90 % of the lumen. This is consistent with our HE staining (Fig. 5A), indicating that the endogenous urothelial progenitor cells are collected from the lumen surface of the scaffold for tissue repair. The difference is that the HA-collagen nanofiber grafts have more Ki67 positive cell layers on both lumen sides than the collagen scaffold grafts. By calculating and comparing Ki67 positive expression levels, we found that the proliferation cell density of the HA-collagen nanofiber graft group was significantly higher than that of the collagen nanofiber graft group (Fig. 5D). These results indicate that M2 phenotype macrophages play a crucial role in the homing process of endogenous urothelial progenitor cells, which is consistent with the published reports that biological scaffolds with appropriate biophysical and biochemical cues can polarize macrophages to M2 phenotype at the wound site, to achieve the state of promoting regeneration [20, 34, 43]. It is well known that in situ tissue regeneration depends on the migration and proliferation of endogenous progenitor stem cells to the wound site [29, 44, 45].

### Tubular HA-collagen nanofibers accelerated in vivo urethral reconstruction process

**Fig. 6:**
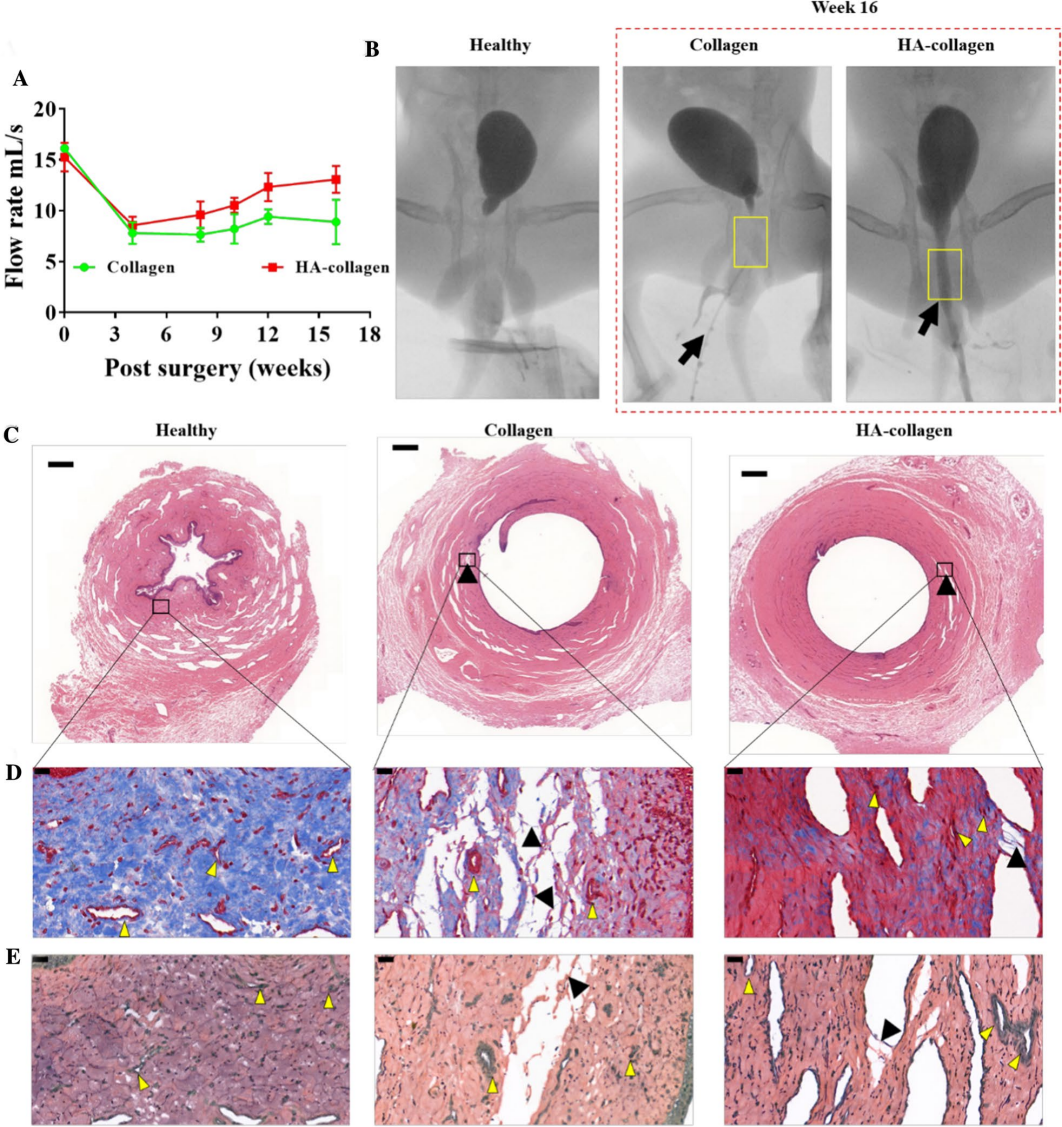
Tubular HA-collagen nanofibers accelerated urethral reconstruction at wound site on week 16 after implantation. **A** The urinary flow rate of the collagen group and HA-collagen group at each predetermined time point after implantation, *n* = 3. **B** Representative voiding cystourethrography of each group on week 16 after implantation. The yellow square areas indicate the position of urethral regeneration. Urocystography of a healthy animal without a urethral graft was used as control sample. The black arrow indicates the discharged contrast agent. Representative **C** HE, **D** Masson’s trichrome, and **E** VVG staining micrographs of the health and regenerated urethras on week 16. The black square areas in **C** (the upper panel) were enlarged in **D**, **E** (the lower panel) at 20$\times$. Scale bars: **C** 350 μm; **D**, **E** 20 μm. Black triangles indicate the location of the degraded nanofibrous scaffold, yellow triangles indicate the blood vessels

To evaluate the functional recovery of the regenerated urethra, uroflowmetry was performed at 4, 8, 10, 12, and 16 weeks after transplantation. The mean uroflow rate curve of the two groups shown in Fig. 6A shows that both the regenerated urethra bridged by tubular collagen and HA-collagen nanofiber grafts remain unobstructed. At 16 weeks after implantation, retrograde cystography showed that after gently pressing the bladder of each animal, the contrast medium could smoothly pass through the regenerated urethras bridged by tubular collagen and HA-collagen nanofibers grafts (Fig. 6B). The regenerated urethra maintained good continuity with the natural urethra, and no complications such as proximal stricture, urinary leakage, and distal stricture were observed. It should be noted that 16 weeks after implantation, the urinary flow velocity (about 15.1 mL/s) of the HA-collagen group was close to that before the urethral trauma (normal), which was higher than that of the collagen group (10.8 mL/s). This indicates that the rapid functional recovery of tubular HA-collagen nanofiber graft group depends on the surface characteristics of tubular HA-collagen nanofiber, that is, the thin HA-coating atop each collagen naofiber. These data suggest that the surface characteristics of biomaterial scaffolds control cell function, which is very important for the rapid recovery of tissue function.

To further determine the long-term effect of macrophage polarization to M2 phenotype on endogenous cell function, the regenerated urethras bridged with tubular HA-collagen and collagen nanofiber grafts were harvested 16 weeks after implantation to observe the tissue regeneration. As shown in Fig. 6C, compared with the healthy urethra, HE staining of the cross-section of the regenerated urethra bridged by tubular HA-collagen nanofiber grafts showed that the uroepithelium showed the same stratification as the original urethra, and the formation of smooth muscle bundles was more extensive. It is worth noting that at low magnification (2.5 $\times$), HE staining of the cross-section of the regenerated urethras bridged with tubular HA-collagen nanofiber graft showed that the tissue regeneration rate matched the degradation rate of tubular HA-collagen nanofibers, while the degradation rate of collagen nanofibers was faster than that of tissue regeneration. To further verify this phenomenon, Masson’s trichrome staining was performed on the same cross-section and then observed at higher magnification (50$\times$). The gradual degradation of HA-collagen and collagen nanofiber could be observed, and the endogenous urine-derived stem cells (they are characterized by positive CD34 [46], Additional file 1: Fig. S7) filled the degraded nanofiber area **(**Fig. 6D). These results suggest that M2 macrophages contribute to the recruitment of endogenous progenitor stem cells. It is consistent with the published reports that anti-inflammatory cytokines promote the rapid recruitment, migration, and infiltration of endogenous cells [30, 47, 48]; promote tissue regeneration and functional healing. In addition, VVG staining of the same cross-section showed that both elastin (black) and blood vessels (yellow) were present in the regenerated urethral tissue, indicating that the endogenous host cells synthesized and deposited new ECM and then infiltrated into the host blood vessels to form a stable vascular network **(**Fig. 6E). These data suggest that urethral grafts made of tubular HA-collagen nanofibers can promote urethral tissue regeneration in situ.

## Conclusions

Here we introduce the design idea and practical application of tubular HA-collagen nanofibers with appropriate biophysical and biochemical clues as hollow organ scaffolds. A layer of HA coating atop each collagen nanofiber surface makes the nanofibrous substrate have higher surface wettability and mechanical softness, which promoted the elongation of macrophages and induced the polarization to M2 phenotype. In addition, elongation promoted the effect of M2 inducing anti-inflammatory cytokines and suppressed the effect of M1 inducing inflammatory cytokines to promote tissue remodeling in situ. One of the main results of this study is to reveal that the design of biomaterial scaffolds for in situ tissue engineering requires precisely controlled surface biophysical signals to guide endogenous cells (including macrophages and progenitor cells) to the injured site for in situ tissue regeneration. In vivo experiments showed that the grafts made of hollow tubular HA-collagen nanofibers could recruit anti-inflammatory M2 macrophages to enrich on its surface, thus producing anti-inflammatory cytokines from local macrophages. The sustained release of these signals can promote the rapid recruitment, migration, and infiltration of endogenous cells and promote tissue regeneration and functional recovery. We used urethra reconstruction as a typical example to demonstrate the concept that we expect the method is applicable to other hollow organ repairs, too. With this approach, we hope to contribute a novel class of biomaterials that adjusts immune responses and recruits endogenous stem cells. The method works very well for urethras, but we expect it would be suitable for broader if situ tissue regeneration applications such as in blood vessels and in gastrointestinal tract reconstruction.

## Supplementary Information

**Additional file 1. Fig. S1. **Scaffold implantation. **Fig. S2. **TGAthermograms of collagen, HA-collagen nanofibrous films and HA. **Fig. S3. **Typicalstress-strain curves of the collagen and HA-collagen nanofibrous films.**Table S1.** Contact water angle andmechanical properties of collagen and HA-collagen nanofibers. **Fig. S4. **Effectivecell adhesion to collagen and HA-collagen nanofibrous films. **Fig. S5. **Invitro cyto-compatibility assay. **Fig. S6. **Double immunofluorescence analysis of regeneratedurethral sections on week 2 after the implantation. **Fig. S7. **Double immunofluorescence analysis of regeneratedurethral sections on week 16 after the implantation

## Data Availability

All data are available in the main text or the additional materials.
