# Supplementary material for: HA-coated collagen nanofibers for urethral regeneration via in situ polarization of M2 macrophages
Source: J Nanobiotechnology. 2021 Sep 22;19:283. doi: 10.1186/s12951-021-01000-5 (PMC8456673; doi:10.1186/s12951-021-01000-5)
Supplement: Supplementary file 1 — Additional file 1. Fig. S1. Scaffold implantation. Fig. S2. TGAthermograms of collagen, HA-collagen nanofibrous films and HA. Fig. S3. Typicalstress-strain curves of the collagen and HA-collagen nanofibrous films.Table S1. Contact water angle andmechanical properties of collagen and HA-collagen nanofibers. Fig. S4. Effectivecell adhesion to collagen and HA-collagen nanofibrous films. Fig. S5. Invitro cyto-compatibility assay. Fig. S6. Double immunofluorescence analysis of regeneratedurethral sections on week 2 after the implantation. Fig. S7. Double immunofluorescence analysis of regeneratedurethral sections on week 16 after the implantation [file 12951_2021_1000_MOESM1_ESM.docx]

# Additional Information (ESI)

HA-coated collagen nanofibers for urethral regeneration via in situ polarization of M2 macrophages

Yuqing Niu^a^, Florian J. Stadler^b^, Xu Yang^a^, Fuming Deng^a^, Guochang Liu^a^, Huimin Xia ^a, *^.

^a.^Department of Pediatric Surgery, Guangdong Provincial Key Laboratory of Research in Structural Birth Defect Disease, Guangzhou Women and Children’s Medical Center, Guangzhou Medical University, Guangzhou 510623, Guangdong, PR China

^b.^Nanshan District Key Lab for Biopolymers and Safety Evaluation, Shenzhen Key Laboratory of Polymer Science and Technology, Guangdong Research Center for Interfacial Engineering of Functional Materials, College of Materials Science and Engineering, Shenzhen University, Shenzhen 518055, PR China


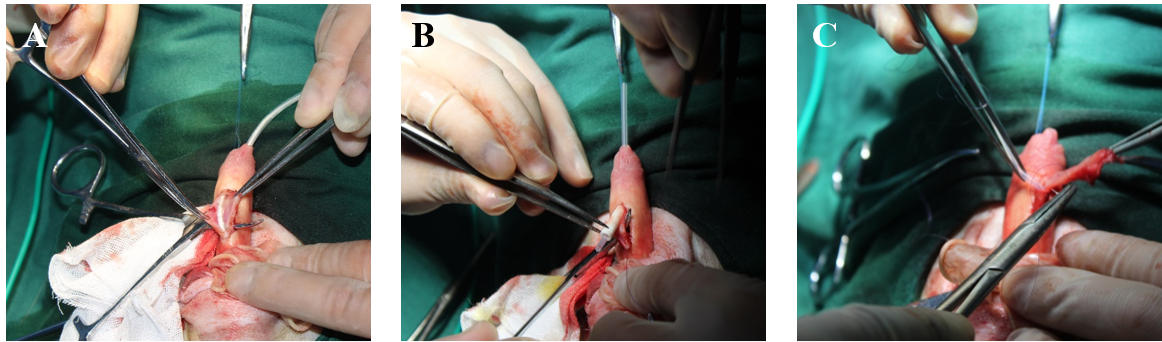


**Fig. S1. Scaffold implantation.** (A) A 2 cm long model of instrumented urethral incision was made with a scalpel and scissors. (B) 6-0 suture was used to suture the host urethral tissue and one end of the tubular nanofiber scaffold (length 2.2 cm). (C) The suture was used to suture the other end of the host urethral tissue.


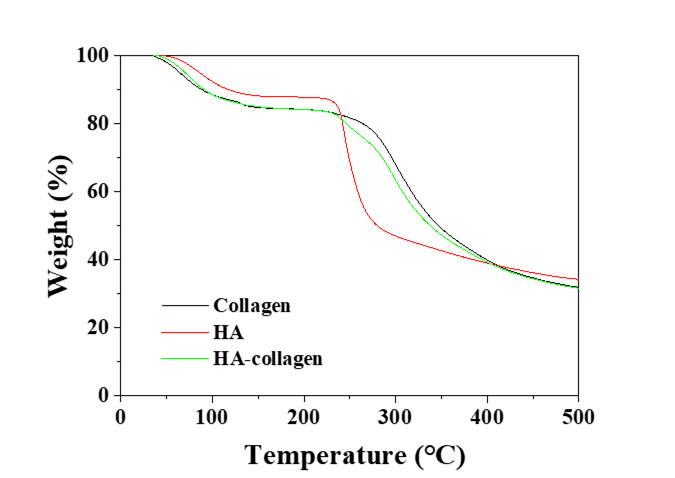


**Fig. S2. TGA thermograms of collagen, HA-collagen nanofibrous films and HA.**


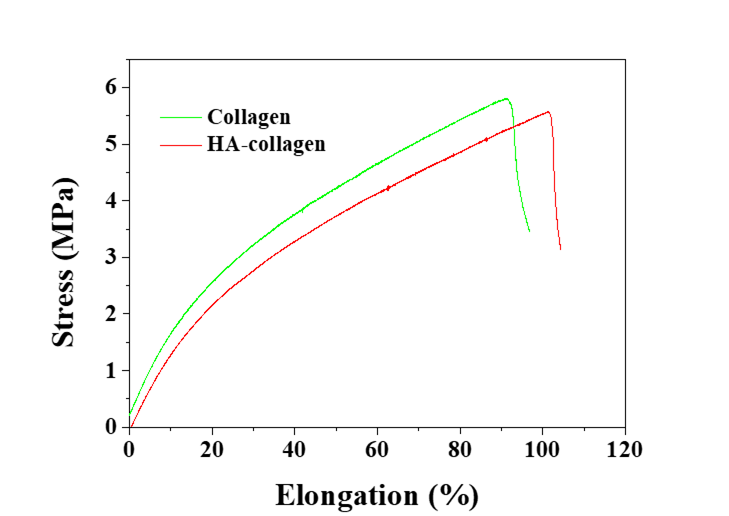


**Fig. S3. Typical stress-strain curves of the collagen and HA-collagen nanofibrous films.**

**Table S1.** **Contact water angle and mechanical properties of collagen and HA-collagen nanofibers.**

| **Sample** | ***θ*H_2_O (°)^a^** | ***E* (MPa)^b^** | ***δ* (MPa)^c^** | ***ε* (%)^d^** |
| --- | --- | --- | --- | --- |
| **Collagen** | 86.5$\pm$6.2 | 0.35$\pm$0.3 | 0.13$\pm$0.3 | 91$\pm$11 |
| **HA-collagen** | 75.2$\pm$1.1 | 0.34$\pm$0.4 | 0.14$\pm$0.1 | 101$\pm$28 |

^a^: Contact water angle.

^b^: Young’s modulus.

^c^: Stress at yield.

^d^: Strain at break.


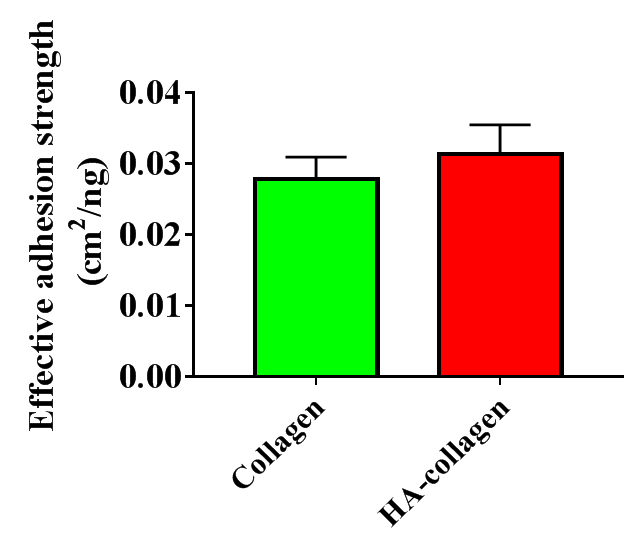


**Fig. S4. Effective cell adhesion to collagen and HA-collagen nanofibrous films.**


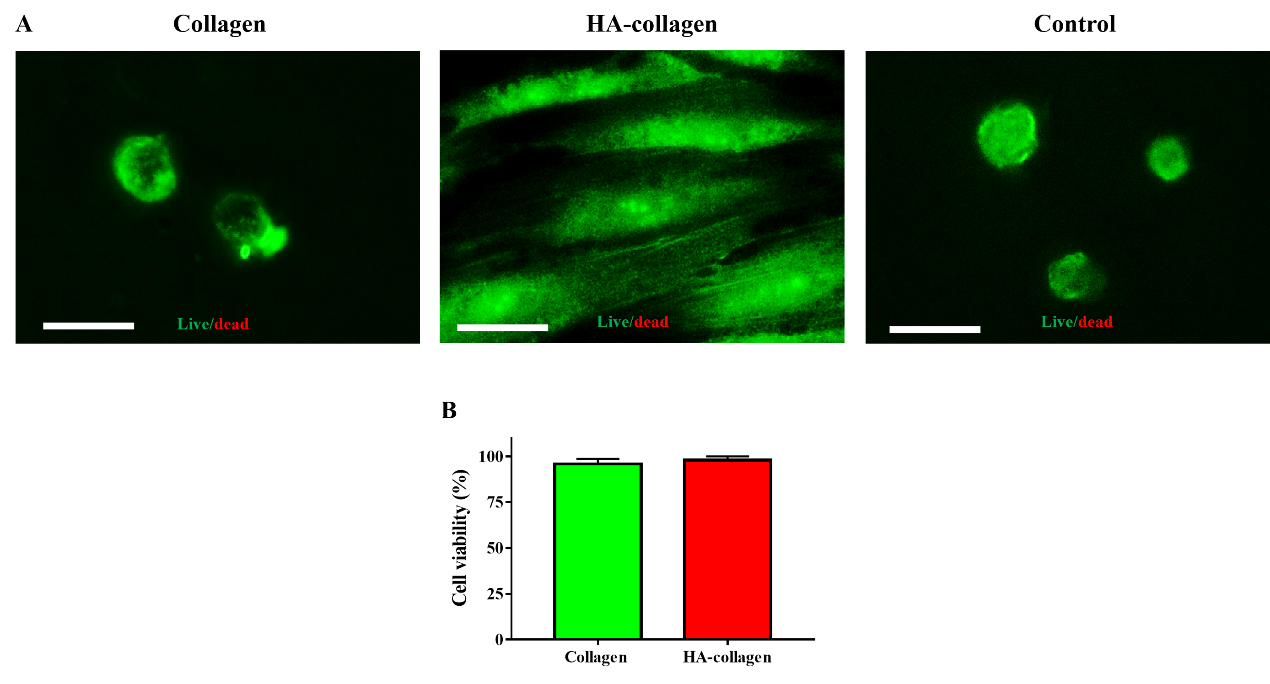


**Fig. S5. In vitro cyto-compatibility assay.** (A) Live/dead stain of macrophages in different nanofibrous scaffolds and cell plate (control) on day 7 after seeding. Scale bar: 20 μm. (B) The quantitative data for cell viability of macrophages in collagen and HA-collagen nanofibrous films on day 7 after seeding.


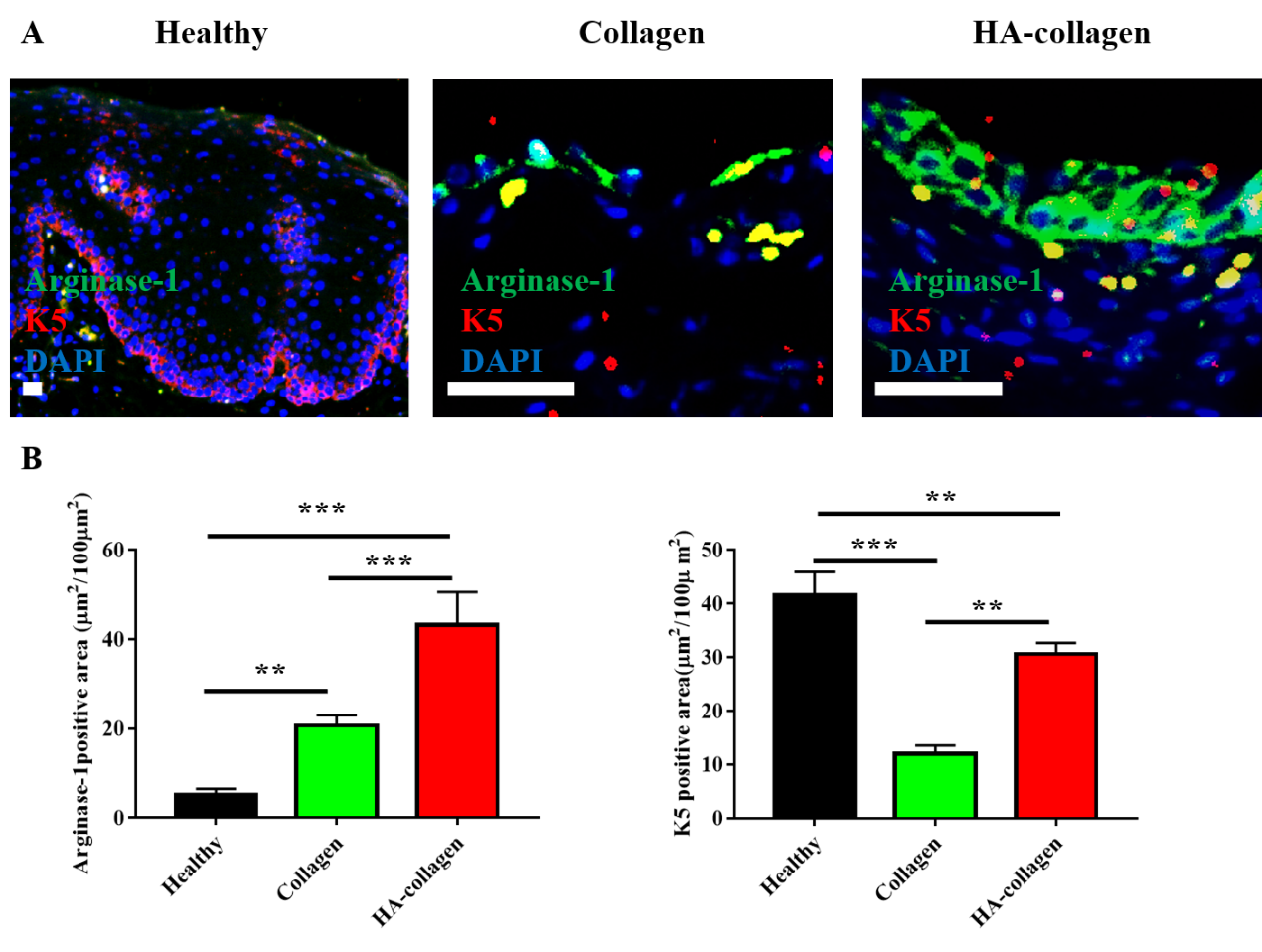


**Fig. S6. Double immunofluorescence analysis of** **regenerated urethral sections on week 2 after the implantation.** (A) Fluorescent staining of the middle cross-section stained for UPC (red), M2 macrophage (green), and nuclei (blue) in the indicated groups at 2 weeks after implantation. Scale bars: 20 μm. (B) Quantification of arginase-1- and K5-positive area per 100 μm^2^ in the indicated groups at week 2 after the implantation (5 random fields per animal, n=3 animal per group). ** p < 0.01, *** p < 0.001.


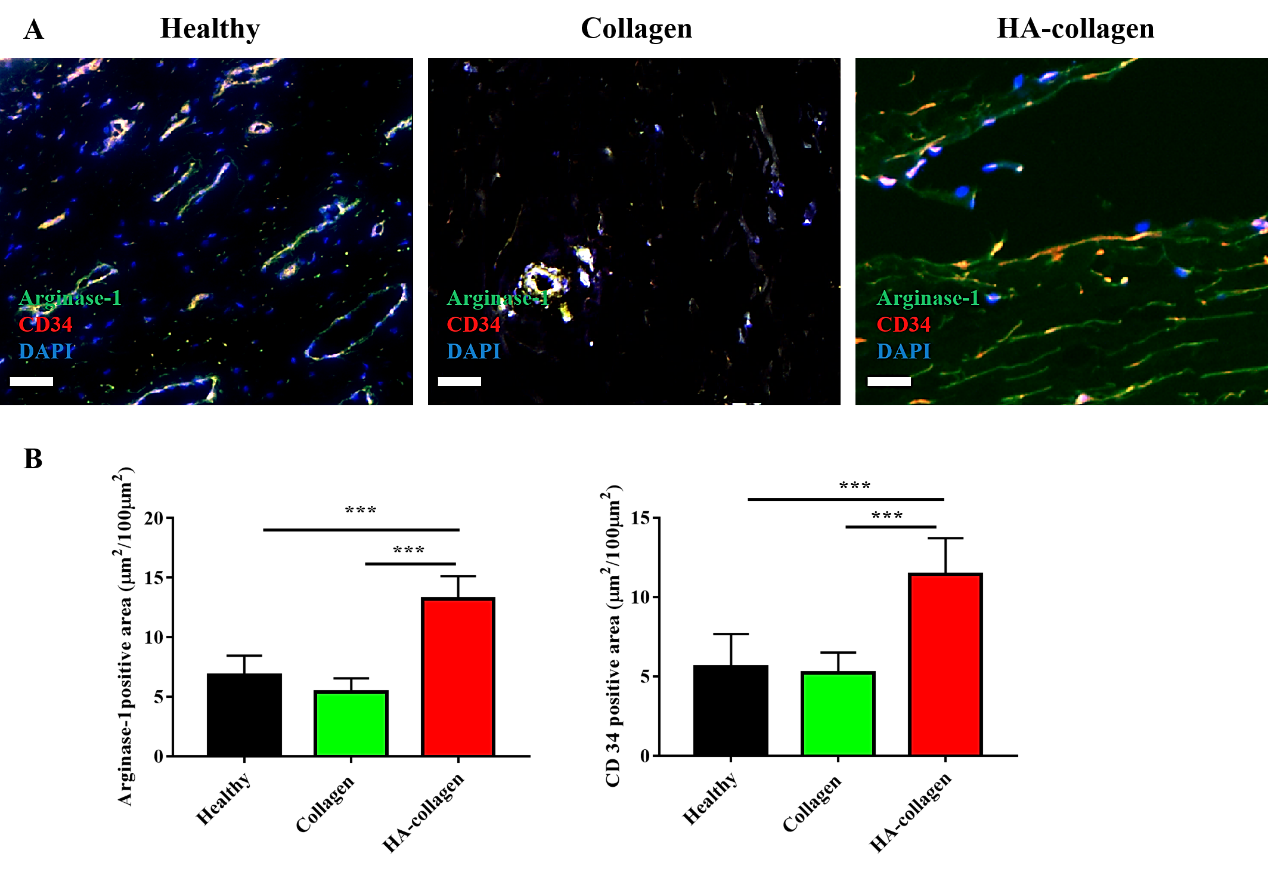


**Fig. S7. Double immunofluorescence analysis of** **regenerated urethral sections on week 16 after the implantation.** (A) Fluorescent staining of the middle cross-section stained for USCs (red), M2 macrophage (green), and nuclei (blue) in the indicated groups at 16 weeks after implantation. Scale bars: 40 μm. (B) Quantification of arginase-1- and CD34-positive area per 100 μm^2^ in the indicated groups at week 16 after the implantation (5 random fields per animal, n=3 animal per group). *** p < 0.001.
